# Acceptability of Minimalist Shoes Compared With Balance‐Enhancing Shoes in Older Women: Protocol for a Randomised Crossover Trial

**DOI:** 10.1002/jfa2.70129

**Published:** 2026-02-11

**Authors:** Ameer Nor Azhar, Shan M. Bergin, Shannon E. Munteanu, Hylton B. Menz

**Affiliations:** ^1^ Discipline of Podiatry, School of Allied Health, Human Services and Sport La Trobe University Melbourne Victoria Australia

**Keywords:** accidental falls, clinical trial protocol, postural balance, shoes

## Abstract

**Introduction:**

Falls are a major concern for older women and can result in significant injury. Footwear has been shown to improve or impair balance performance in older women, contingent upon footwear design features. Balance‐enhancing shoes may reduce the risk of falling but their acceptability is unknown. Acceptability is important because it influences the level of adherence to the intervention. The aim of this trial protocol is to describe the methodology of a randomised crossover trial to compare the acceptability of balance‐enhancing outdoor shoes versus minimalist outdoor shoes in older women and compare the effects of these two interventions on the perceived risk of falling and balance performance.

**Methods:**

The trial will use a randomised two‐period crossover trial methodology. We will recruit 44 community‐dwelling women aged 65 years or older who will be randomised to receive a pair of minimalist shoes (Basic Lace Up Canvas Shoes, Kmart Australia Ltd, Mulgrave, Australia) or a pair of balance‐enhancing shoes (Balla Balance Leather Lace Up Boots, Ziera Australia, Abbotsford, Australia), which encompass key features known to be beneficial for balance such as adequate fixation, a firm heel counter, high heel collar, firm midsole and textured insole. The order of the interventions will be randomised. Cross‐over to the second shoe condition will occur at 6 weeks. Outcome measures will be collected at baseline, six and 12 weeks; the primary endpoint for assessing footwear acceptability for each shoe condition will be 6 weeks. The primary outcome measure will be footwear acceptability, evaluated using a modified version of the Monitor Orthopaedic Shoes Questionnaire. Secondary outcome measures include perceived risk of falling (the Falls Efficacy Scale International) and balance performance (upper body stability when walking, using the GyKo wearable sensor).

**Discussion:**

This trial will evaluate the acceptability, perceived risk of falling and balance performance of minimalist shoes versus balance‐enhancing shoes. The findings will provide much‐needed evidence as to the acceptability of these two shoe types in older women. Such information may support footwear design to increase balance performance and reduce risk of falling.

**Trial Registration:**

Australian and New Zealand Clinical Trial Registry (ACTRN12624001496505)

## 1 Background

One in three community dwelling older adults will experience a fall each year [1], with older women having a higher incidence of falling compared with older men due to anatomical and physiological differences and age‐related changes such as menopause [2]. Older women also have an increased risk of significant fall‐related injury, such as hip fracture or head trauma, compared with men [3]. Evidence also indicates that older women have a greater intrinsic fear of falling compared with older men [4], so it is important to address and mitigate the risk of falls in this population.

Footwear is a modifiable factor that has been shown to influence balance performance and gait. Specific characteristics of footwear, such as elevated heel height, poor fit, inadequate fixation and soft midsoles impair balance performance in older people. Conversely, features such as a high ankle collar, adequate fixation and firm‐to‐hard density midsoles are beneficial to balance performance [5, 6, 7, 8]. However, few commercially available shoes meet all these requirements.

There are two main approaches to optimise balance: (i) minimalist shoes and (ii) balance‐enhancing shoes. Minimalist shoes are thought to optimise balance by allowing the stimulation of plantar mechanoreceptors, whereas balance‐enhancing shoes provide greater support and encompass features thought to improve balance [6]. However, the relative merit of these approaches is unclear. Although minimalist shoes have been found to be more effective for balance than going barefoot [9] or wearing conventional shoes [10], there is no significant difference in the immediate effects of minimalist shoes and balance‐enhancing shoes [11]. Furthermore, as minimalist shoes tend to be less durable, their effects may diminish over time.

Acceptability of footwear is an important factor. A shoe's acceptability is influenced by multiple factors that include how aesthetically pleasing it is to the user and their perceptions of others' views, ease of use and level of comfort [12, 13]. If a shoe not considered acceptable, it may reduce adherence, reducing the potential benefit to the user [14]. The purpose of this trial protocol is to describe the methodology of a randomised crossover trial to compare the acceptability, perceived risk of falling and balance performance of balance‐enhancing outdoor shoes versus minimalist outdoor shoes in older women. Participants will be allocated to each condition for 6 weeks.

## 2 Methods

This study protocol has been reported using the Standard Protocol Items: Recommendations for Interventional Trials (SPIRIT) guidelines [15], and the associated checklist is included as Supporting Information S1. Publications associated with the trial will be reported according to the Consolidated Standards of Reporting Trials (CONSORT) 2010 Statement extension for randomised crossover trials [16]. The trial has been registered with the Australian New Zealand Clinical Trials Registry (ACTRN12624001496505).

### 2.1 Ethical Approval

Ethical approval has been obtained from the La Trobe University Human Research Ethics Committee (HEC24476). All participants will provide informed consent prior to being recruited into the trial.

### 2.2 Design

**FIGURE 1:**
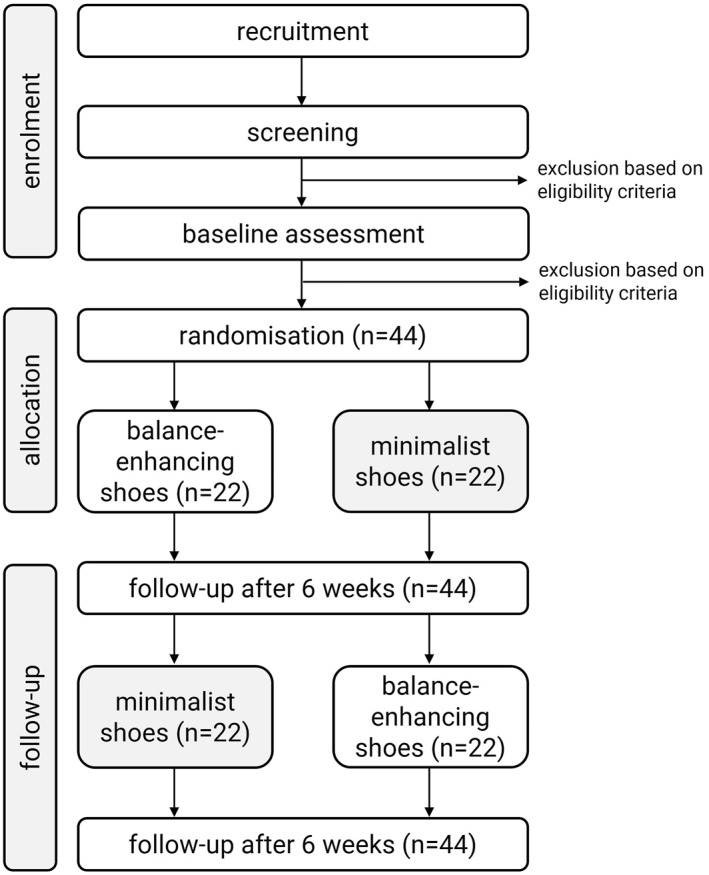
Trial profile.

This study (BALANCER‐OUT: BALAnce eNhanCing shoEs for oldeR women: OUTdoor) is a randomised crossover trial which will take place over a 12‐week period (each intervention period will be 6 weeks). Participants will be provided with both shoe types (minimalist and balance‐enhancing) with the order of wear randomised (participant intervention order will be AB or BA). Crossover to the alternate shoe condition will take place at the end of week 6. Although commonly used in crossover trials, we have deemed that a wash‐out period is not required in this trial as we have determined that any carry‐over effect would be minimal, and the outcome measures are specific to the intervention(s) being applied (i.e., we are examining the acceptability of the footwear as the primary outcome). Our approach is supported by a recent crossover trial comparing two types of shoes for older people with foot pain that did not employ a wash‐out period between interventions [12]. Figure 1 depicts the trial profile and the timing of enrolment, interventions and assessments are shown in Table 1. The study design and nature of the interventions preclude participants and researchers administering the intervention from being blinded to group allocation. However, trial personnel entering participant‐reported data and the biostatistician performing the statistical analyses for the trial will be blinded. Assessments will be performed at the Foot and Ankle Laboratory at La Trobe University, Bundoora (Victoria, Australia).

**TABLE 1:** SPIRIT (Standard Protocol Items: Recommendations for Interventional Trials) diagram of enrolment, interventions and assessments.

	Study period			
	Enrolment	Allocation	Post‐allocation	
Timepoint	*t*_0_	*t*_1_	*t*_2_	*t*_3_
Enrolment				
Eligibility screen	X			
Informed consent	X			
Sizing of shoes	X			
Baseline assessments				
Demographics		X		
General health questionnaire		X		
QuickScreen questionnaire		X		
Monitor orthopaedic shoes questionnaire		X		
Falls efficacy scale international questionnaire		X		
Balance tests using GyKo sensor		X		
Allocation		X		
Interventions				
Balance‐enhancing shoes followed by minimalist shoes		X		
Minimalist shoes followed by balance‐enhancing shoes		X		
Outcome assessments				
Monitor orthopaedic shoes questionnaire			X	X
Falls efficacy scale international			X	X
Balance tests using GyKo sensor			X	X
Credibility and expectancy questionnaire		X	X	
Other assessments				
Adherence			X	X
Adverse events			X	X

*Note: t*_0_ = week 1, *t*_1_ = week X, *t*_2_ = week X + 6, *t*_3_ = week X + 12. NB: The time from fitting appointment (*t*_0_) to intervention allocation (*t*_1_) is contingent on availability of footwear and it is estimated to be anywhere between two to 3 weeks.

### 2.3 Participant Recruitment and Eligibility Criteria

Participants will be recruited via a postal invitation using a database of patients from the La Trobe University Health Sciences Clinic and social media advertising. Forty‐four participants will be recruited for the trial. To be eligible, participants must (i) identify as a woman, (ii) be aged ≥ 65 years, (iii) be able to walk household distances (more than 50 m) without the use of a walking aid, (iv) be free of any disease or condition which interferes with balance or walking (e.g., Parkinson's disease), (vi) be free of any lower limb or partial foot amputation, (vii) be free of any surgery to the foot or ankle in the past 3 months and (viii) be willing to wear two different types of footwear across two 6‐week periods.

Participants will be measured for their shoe size using the Brannock device [17]. This will take place during an initial fitting appointment to ensure that participants are initially comfortable and satisfied with the shoe size they will be allocated during the trial. Participants will attend the initial appointments four to 5 weeks prior to the baseline assessment to allow for the shoes (both minimalist and balance‐enhancing) to be ordered from their respective suppliers.

### 2.4 Baseline Assessment

Participant characteristics (such as age, education and ethnicity), major medical conditions and number of medications will be obtained via an online questionnaire designed in a secure web platform (REDCap, Research Electronic Data Capture, Vanderbilt University, USA) [18, 19]. Height and weight will be measured using a stadiometer and digital scales and body mass index will be calculated as weight (kg)/height (m^2^). Foot pain will be measured using the Manchester‐Oxford Foot Questionnaire [20] and overall health will be measured using the 5‐level EuroQOL‐5D version (EQ‐5D‐5L) [21]. Shoe acceptability, perceived risk of falls and balance performance will also be measured (described later).

Risk of falling will be evaluated using the validated QuickScreen tool [22], which consists of 8 parameters: (i) previous falls, (ii) total medications, (iii) use of psychotropic medications, (iv) visual acuity (using a 10% low contrast letter chart), (v) touch sensation (using a Semmes–Weinstein‐type pressure aesthesiometer applied to the lateral malleolus), (vi) the sit to stand test (using a 430 mm high chair without armrests, 5 times as fast as possible with arms folded), (vii) the near tandem stand test (eyes closed, with feet separated laterally by 25 mm and the heel of the front foot 25 mm anterior to the great toe of the back foot) and (viii) the alternate step test (alternatively placing the whole left and right feet as fast as possible onto a 190‐mm high and 400‐mm deep step 8 times). Each of these measures will be dichotomised using established cut‐points [22].

### 2.5 Randomisation

The order of the interventions (minimalist vs. balance‐enhancing shoes) will be randomised (i.e., AB or BA) using an online randomisation service (www.sealedenvelope.com). A third‐party individual who is not involved in the study design, recruitment and data analysis will create and upload the allocation sequence to REDCap. REDCap's randomisation feature allows for allocation concealment by enabling different user rights. Assessors involved in recruitment and data collection will not have user rights to access the uploaded allocation sequence. All baseline assessments will be completed prior to intervention order allocation.

### 2.6 Interventions

**FIGURE 2:**
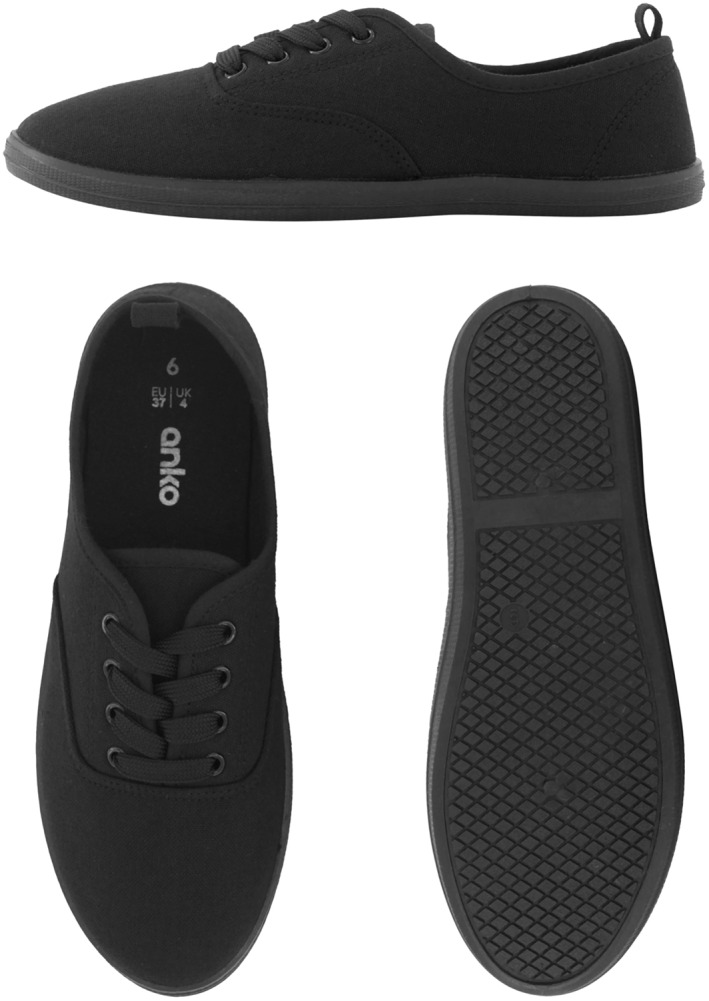
Textured insole for the balance‐enhancing shoe.

**FIGURE 3:**
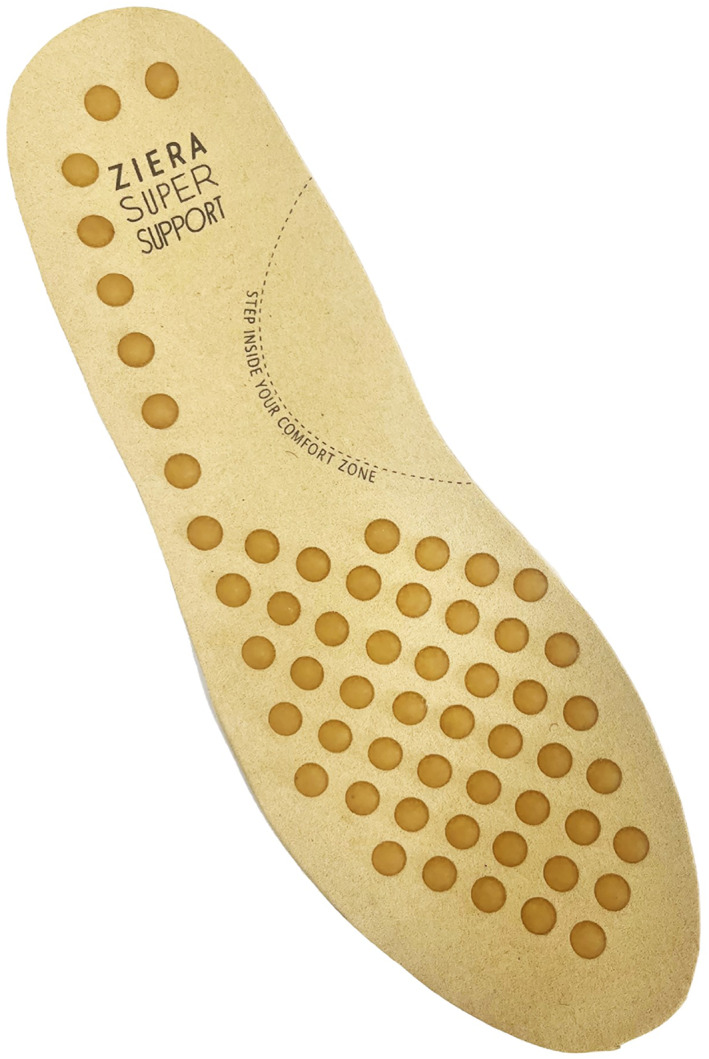
Minimalist shoe.

**FIGURE 4:**
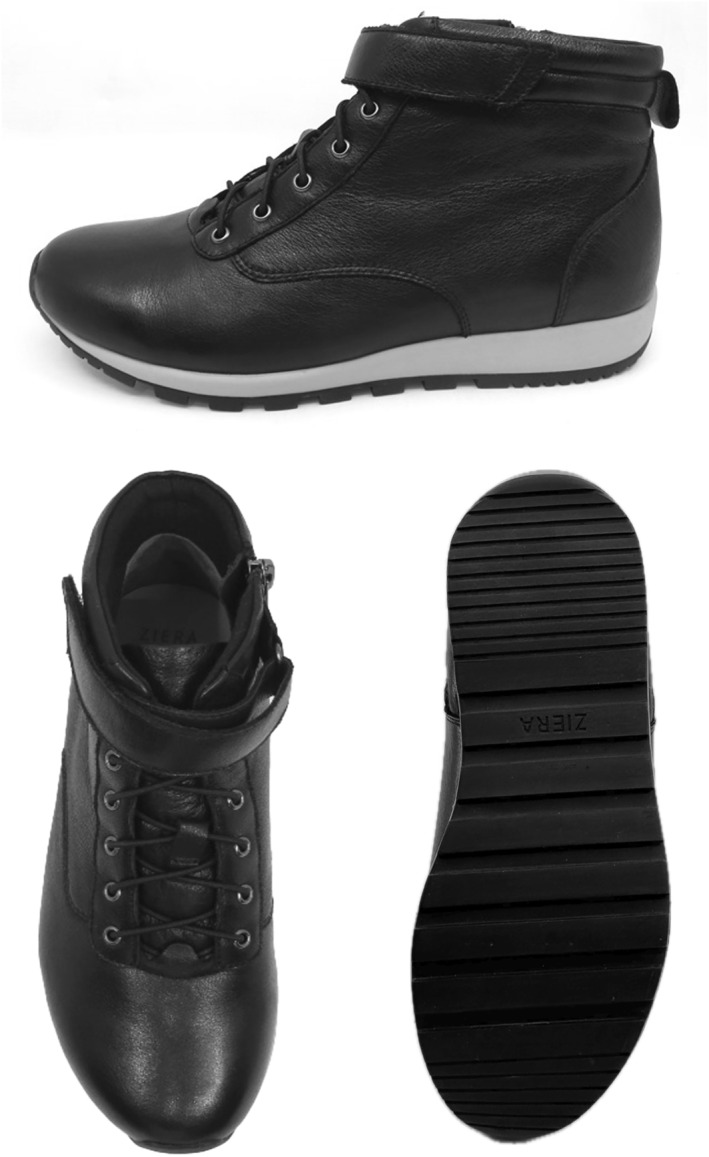
Balance‐enhancing shoe.

Participants will be required to wear two different types of shoes (minimalist and balance‐enhancing) over a 12‐week period (6 weeks in each shoe condition). Participants will be asked to wear both shoe types as often as possible throughout the study period when outdoors. For the minimalist shoe condition, participants will be provided with Basic Lace Up Canvas Shoes (Kmart Australia Ltd, Mulgrave, Australia), which meet the definition of minimalist shoes by Esculier et al. [23], namely that they provide ‘minimal interference with the natural movement of the foot due to its high flexibility, low heel to toe drop, weight and stack height and the absence of motion control and stability devices’. The minimalist shoes have a canvas upper and rubber sole with lace fixation. The weight of the minimalist shoe is 191–258 g across the size range and similar shoes have been used for other comparable studies [11]. See Figure 2. For the balance‐enhancing footwear condition, participants will be provided with Balla Balance Leather Lace Up Boots (Ziera Australia, Abbotsford, Australia), which include various balance‐enhancing features such as (i) increased collar height, (ii) adequate fixation (iii) firm midsole and (iv) textured insoles (as seen in Figure 3) [6]. The footwear has a firm (Shore A hardness 55) [24] rubber sole of 20 mm thickness under the heel and 10 mm under the forefoot, laces plus Velcro fastening, and a firm heel counter. The outsole has a 10° bevel into the heel region [25, 26], grooves perpendicular to the sole (1.2 mm deep and 2.4 mm wide) across the heel surface area [27] and perpendicular grooves (5 mm deep and 12 mm wide) across the rest of the sole [28, 29]. The balance‐enhancing shoe also includes a textured insole constructed from 4 mm thick ethyl vinyl acetate (Shore A 25) [24] with approximately 56 (total number may vary due to size) dome‐shaped projections (3 mm high and 8 mm diameter, Shore A 85) [24] placed across the forefoot in a 15‐mm diamond pattern and along the lateral border, extending to the heel. The design of the textured insole is informed by previous studies reporting improvements in balance when similar insoles are worn [11, 30, 31, 32]. The weight of the balance‐enhancing shoe is 328–430 g across the size range. See Figure 4. Participants will be able to continue their normal indoor footwear wearing habits when indoors during the trial.

### 2.7 Treatment Credibility/Expectation

Treatment credibility (belief about the logic underpinning the intervention) and expectation (perception of how much they may benefit) will be assessed using the Credibility/Expectancy Questionnaire (CEQ) [33]. The CEQ will be administered after the allocation of both shoe conditions (at baseline and week 6). The CEQ consists of 6 items and for each item; participants will be asked to rate the credibility of the intervention and their expectations on a 9‐point Likert scale. High scores on the scale indicate that the participant considers the intervention to be credible and expects it to be effective. As the CEQ was originally intended for people receiving a form of treatment, to ensure it is appropriate for the trial, the wording of the 6 items in the CEQ will be modified to focus on the footwear and how it pertains to balance.

### 2.8 Outcome Measures

The primary outcome measure is footwear acceptability [13], which will be measured using a modified Monitor Orthopaedic Shoes Questionnaire (MOSQ) at weeks 6 and 12, which allows participants to have 6 weeks in each shoe condition. The MOSQ consists of seven items which address attractiveness to self, attractiveness to others, comfort, fit, ease of donning and doffing, weight satisfaction and perceived durability. All items are scored on a 100‐point VAS. Each of the 7 MOSQ items will be reported separately, but our primary outcome will be calculated from the average score of the 7 items (attractiveness to self, attractiveness to others, comfort, fit, ease of donning and doffing, weight and durability).

Secondary outcome measures will be perceived risk of falling using the Falls Efficacy Scale International (FES‐I) [34] and balance performance (upper body stability when walking, using the validated GyKo sensor) [35, 36], both measured at weeks 6 and 12. The FES‐I is a widely accepted tool to assess an individual's concern about falling [37], which asks about 16 activities and uses a 4‐point Likert scale with the following options: not at all concerned, somewhat concerned, fairly concerned and very concerned. The minimum score is 16 (no concern about falling) and the maximum score is 64 (severe concern about falling). Both the MOSQ and FES‐I will be administered as surveys using REDCap.

Balance performance will be determined from the upper body sway when walking using the GyKo sensor (dimensions: 50 × 70 × 20 mm; mass: 35 g; GyKo, Microgate, Bolzano, Italy). The sensor will be attached to participants at the height of the thoracic spine using a proprietary harness. The sensor can document movements up to 16 g and angular velocities of up to 2000°/sec with an acquisition frequency of 1000 Hz. Participants will be asked to walk over an 8‐m‐long irregular surface (foam plates randomly placed and covered with artificial grass) at their own comfortable speed. An irregular surface will be employed to replicate real outdoor walking conditions as surfaces which are smooth and flat are less common. This approach has been used previously and provides better discrimination of falls risk than walking on a level surface [38]. Three upper body parameters will be measured (i) total length (measured in millimetres, defined as the total excursion of the sway path), (ii) antero‐posterior length (measured in millimetres, defined as the total excursion in the antero‐posterior direction) and (iii) medio‐lateral length (measured in millimetres, defined as the total excursion in the medio‐lateral direction). These measures will be divided by walking speed (measured as metres/second, so 8 m divided by the duration of each trial in seconds) as walking speed is known to affect stability measures [39, 40]. Four trials will be recorded, and the average will be calculated.

### 2.9 Preference

Preference for either shoe condition will be captured at the end of the trial for each participant. A REDCap survey will be created to ask participants which shoe condition (minimalist or balance‐enhancing) was preferred or if there was no preference.

### 2.10 Evaluation of Adherence

Adherence will be assessed using the Orthotimer temperature sensor. The Orthotimer system is a valid and reliable instrument, which objectively measures adherence to wearable devices including orthoses and footwear [41]. The sensor will be embedded into both types of shoe styles (minimalist and balance‐enhancing) in locations imperceptible to participants. According to the manufacturer, the sensor has quartz‐controlled time measurement at 32,768 kHz frequency and a temperature precision of ± 0.1°C. The sensor dimension is 9 × 13 × 4.5 mm. Each participant will be allocated one Orthotimer sensor and it will be placed in the shoe (minimalist or balance‐enhancing) that the participant is allocated to at that timepoint. The sensors will be placed into shoes prior to the shoes being issued to participants, and the data will be collected during each 6‐week period. The Orthotimer sensors will be set to capture data at 20‐min intervals.

### 2.11 Adverse Events

Adverse events will be reported as the number of participants experiencing any adverse event (serious or nonserious), the number and types of adverse events and the number of withdrawals because of adverse events. Adverse events will be documented at weeks 6 and 12 via a REDCap survey [42]. Participants will be asked to document the type of adverse event, the body location and the severity of the effect. Serious adverse events will be defined as events that are life‐threatening, require hospitalisation or result in persistent or significant disability or incapacity [43]. Participants experiencing a serious adverse event (e.g., significant pain) will be asked to contact one of the investigators and further management will be implemented as appropriate.

### 2.12 Data Management

Enrolment, baseline and follow‐up questionnaire data will be stored in REDCap and subsequently stored as electronic files on the La Trobe University secure server.

### 2.13 Sample Size

In crossover trials, each participant acts as their own control, which greatly reduces between‐participant variability and therefore reduces the required sample size. In the absence of a widely used primary outcome measure, we averaged data from five questions from the MOSQ [13] used in our recent pilot study [11]. These questions are related to participant perceptions of the attractiveness (to both self and others), comfort, fit and ease of donning and doffing of minimalist shoes versus balance‐enhancing shoes, scored on a 0 to 100 visual analogue scale. Using the crossover trial formula described by Jones and Kenward [44], a mean difference of 12.5, standard deviation of 27.8, power of 0.80 and alpha level of 0.05, we determined that a total of 39 participants would be required. This equates to an effect size of 0.45, which is considered a medium effect size using the thresholds proposed by Sawilowsky [45]. Our key secondary outcome measure, the FES‐I [34], has a mean difference of 8.0, and standard deviation of 15.6 when comparing participants with a low and high concern about falling. Using the same formula, we determined that a total of 30 participants would be required, which equates to a medium effect size of 0.51 [45]. To account for an estimated 10% attrition rate, we will recruit an additional 5 participants. Therefore, we will recruit 44 participants to address the sample size requirements for both our primary and key secondary outcome measures.

### 2.14 Statistical Analysis

Statistical analysis will be performed using the most recent version of SPSS (IBM Corp., Armonk, NY, USA) available at the time of analysis. Analysis will adhere to the intention‐to‐treat principle for all randomised participants. Standard tests to assess continuous data for normal distribution will be used and transformation carried out if required. The primary outcome will be averaged data from 5 questions from the MOSQ [13]. Analysis will be performed using a repeated measures analysis of variance. Four effects are worthy of consideration: (i) the treatment effect, (ii) the order effect, (iii) the treatment‐order interaction effect and (iv) the carry‐over effect [46]. The treatment effect is the direct effect of minimalist versus balance‐enhancing shoes. The order effect implies that the sequence in which treatments are given influences the outcome. The treatment‐order interaction effect implies that the effect of the treatment is influenced by the order in which it is presented. Finally, the carry‐over effect refers to the effect of the previous treatment on the next treatment. In our study, we consider the carry‐over effect to be negligible, as the outcome measures are specific to the shoe being worn. Multiple imputation will be used to replace any missing data using 5 iterations, with baseline scores and order as predictors. Frequency of adverse events will be compared using relative risk and risk difference statistics.

## 3 Discussion

The primary aim of this randomised crossover trial is to evaluate the acceptability of minimalist shoes compared with balance‐enhancing shoes in older women. Our recent scoping review [6] identified no fully powered randomised controlled trials that had evaluated acceptability for footwear designed to improve balance, meaning our study will be the first appropriately powered randomised crossover trial to investigate the acceptability of balance‐enhancing footwear.

This trial has multiple strengths. First, to reduce the risk of bias, an external randomisation service for sequence generation will be employed, with the crossover study methodology ensuring each participant acts as their own control. Second, there will be an objective measurement of participant adherence to both interventions using the Orthotimer sensor. Third, the trial will use a follow‐up period of 6 weeks for each intervention, whereas most studies investigating the effects of footwear, including balance‐enhancing shoes, have evaluated immediate effects only [6]. Finally, adherence to wearing each intervention will be maximised by the timing of recruitment and data collection, which avoids the busy and hot months across the Australian summer (December–January).

There are several limitations of this trial. First, the inability to blind both the participants and researchers administering the interventions may introduce performance bias; however, this is a commonly recognised limitation in trials which employ nonpharmacologic interventions [47]. Second, our trial is restricted to women only. This is due to the balance‐enhancing footwear being available only in women's styles and sizing; thus, the results of the trial will not necessarily be generalisable to men. Third, a related issue is that participants will be recruited from a single University Podiatry Clinic. Although the clinic is open to the public and anyone can receive podiatric care, our sample may not be broadly generalisable. Fourth, risk of falls was not included as part of the eligibility criteria for either study, meaning the study sample may include participants at relatively low risk of falls. This decision was intentional and pragmatic as both the footwear conditions are retail products that are commercially available, and the balance‐enhancing shoes are not specifically marketed to older women at elevated risk of falls. Fifth, the MOSQ was developed and validated for footwear acceptability of orthopaedic shoes specifically and includes further measures specific to prosthetics and orthotics. We have opted to use a modified version of this tool as there is currently no tool available for evaluating acceptability of nonorthopaedic, commercial footwear. Lastly, due to the nature of the trial and the interventions we are employing, there may be concerns surrounding the aesthetics of both shoe conditions (minimalist or balance‐enhancing). If participants dislike the design of either shoe condition, this may be a deterrent for intervention adherence.

Findings from the trial will provide much needed data regarding footwear acceptability and balance performance in older women. The results of this trial may be used to inform the design of new footwear promoting better adherence to wearing footwear that may reduce the risk of falls in older people. A qualitative study will also be conducted after the completion of this trial which will focus on the lived experience of women aged 65 years or older who have worn balance‐enhancing shoes.

### 3.1 Trial Status

Recruitment for the trial has commenced and the results are expected to be available in December 2026. Results will be published in an open access repository.

## Author Contributions

**Ameer Nor Azhar:** conceptualization, methodology, writing – original draft, writing – review and editing. **Shan M. Bergin:** conceptualization, methodology, supervision, writing – review and editing. **Shannon E. Munteanu:** conceptualization, methodology, supervision, writing – review and editing. **Hylton B. Menz:** conceptualization, methodology, supervision, writing – review and editing.

## Funding

Ameer Nor Azhar is supported by an industry scholarship co‐funded by La Trobe University and Ecnalabs Pty Ltd. The balance‐enhancing footwear will also be provided by Ecnalabs Pty Ltd.

## Ethics Statement

The trial has obtained approval from the La Trobe University Human Research Ethics Committee (HEC24476) and all participants will provide written informed consent.

## Consent

The authors have nothing to report.

## Conflicts of Interest

Ecnalabs Pty Ltd developed the balance‐enhancing shoe based on designs by Prof Hylton B. Menz and are co‐funding Ameer Nor Azhar's PhD. However, they have played no role in the design of the project and will play no role in the collection of data, analysis, interpretation of results or decision to publish.

## Supporting information


Supporting Information S1

## Data Availability

All unidentifiable data can be accessed from the author on reasonable request.
